# Supplementary material for: Effects of using deep learning to predict the geographic origin of barley genebank accessions on genome–environment association studies
Source: Theor Appl Genet. 2025 Aug 12;138(9):211. doi: 10.1007/s00122-025-05003-w (PMC12343745; doi:10.1007/s00122-025-05003-w)
Supplement: Supplementary file 1 — (pdf 9246 KB) [file 122_2025_5003_MOESM1_ESM.pdf]

Supplementary Material

Effects of using deep learning to predict the geographic origin of barley genebank accessions on genome-environment association studies

Che-Wei Chang[1], and Karl Schmid[1]

[1]University of Hohenheim, Stuttgart, Germany

SLiM simulation

Mutational effect of QTLs

In our simulation, we assumed that the environmental variables of all sites are located in a 95% interval of the expected genetic variation. The expected genetic variation is calculated as $\sigma_g^2 = 2N\sigma_{qtl}^2$, where N is the number of QTLs ($N = 100$) and 2 is for diploidy. Let the boundary of the 95% interval of the expected genetic variation be $\pm c\sigma_g$, where c is a constant, and let the most extreme environmental variable after centering be x. With our assumption, $|x|$ should be equal to or less than $c\sigma_g$. Therefore, we have $c\sigma_g \geq |x|$. We can rewrite it as $c\sqrt{2N\sigma_{qtl}^2} \geq |x|$, and it gives $\sigma_{qtl} \geq \sqrt{\frac{|x|^2}{2Nc^2}}$. With the equation above, we set $\sigma_{qtl} = 0.45$.

Plasticity of selection

We determined the plasticity, the standard deviation (SD) of fitness bell curve, based on the environmental contrasts between connected sites. This was done by calculating the absolute difference between the environmental variables of the two connected sites, denoted as $C_{env} = |Env_i - Env_j|$, where site i is a connected neighbor of the site j with gene flow.

To simulate a sufficiently strong isolation by environment, we assumed that 90% of C_{env} values fall within a 95% interval of the fitness bell curve. Among the selected 312 sites, we found that 90% of C_{env} values were less than 5.7. To set the plasticity parameter ($\sigma_{plasticity}$) for the SLiM simulation, we chose a value of 2.85, such that $2\sigma_{plasticity} = 5.7$, approximately covering the 95% interval under a normal distribution.

Table S1 (Che-Wei:) Number of overlapping significant SNPs detected by different approaches. Rows and columns with *GEA* and *GEAplus* prefix display the results from GEA and GEAplus with principal components of environmental variables. Rows and columns labeled *GeoRef_SPA* and *PredOrigin_SPA* show the results of spatial ancestry analysis (SPA) conducted using geo-referenced accessions and the entire landrace collection with predicted origins, respectively.

	GEA_PC1	GEA_PC2	GEA_PC3	GEAplus_PC1	GEAplus_PC2	GEAplus_PC3	GeoRef_SPA	PredOrigin_SPA
GEA_PC1	709							
GEA_PC2	0	0						
GEA_PC3	0	0	0					
GEAplus_PC1	0	0	0	0				
GEAplus_PC2	27	0	0	0	318			
GEAplus_PC3	0	0	0	0	0	2		
GeoRef_SPA	0	0	0	0	28	0	435	
PredOrigin_SPA	3	0	0	0	0	0	118	435

Table S2 Significant SNPs overlapping between SPA and GEA analyses. Columns with *GEA* and *GEAplus* prefix display the results from GEA and GEAplus with principal components of environmental variables. Columns labeled *GeoRef_SPA* and *PredOrigin_SPA* show the results of SPA conducted using geo-referenced accessions and the entire landrace collection with predicted origins, respectively. A SNP is indicated with "Yes" if it is significant in a certain analysis.

CHROM	POS	GEA_PC1	GEAplus_PC2	GeoRef_SPA	PredOrigin_SPA
2	211638657	No	Yes	Yes	No
2	213288790	No	Yes	Yes	No
2	213545685	No	Yes	Yes	No
2	213881504	No	Yes	Yes	No
2	213992338	No	Yes	Yes	No
2	214018238	No	Yes	Yes	No
2	214136741	No	Yes	Yes	No
2	226273448	No	Yes	Yes	No
2	228217120	No	Yes	Yes	No
2	228312980	No	Yes	Yes	No
2	228312981	No	Yes	Yes	No
2	229391487	No	Yes	Yes	No
2	229866651	No	Yes	Yes	No
2	235507882	No	Yes	Yes	No
2	235584837	No	Yes	Yes	No
2	248560889	No	Yes	Yes	No
2	301663368	No	Yes	Yes	No
2	307366318	No	Yes	Yes	No
2	312493140	No	Yes	Yes	No
2	321483301	No	Yes	Yes	No
2	329047243	No	Yes	Yes	No
2	330527918	No	Yes	Yes	No
2	351175911	No	Yes	Yes	No
4	13207090	No	Yes	Yes	No
4	13207141	No	Yes	Yes	No
4	25069898	No	Yes	Yes	No
4	25070425	No	Yes	Yes	No
4	25830588	No	Yes	Yes	No
5	7574591	Yes	No	No	Yes
5	7574623	Yes	No	No	Yes
5	17002730	Yes	No	No	Yes

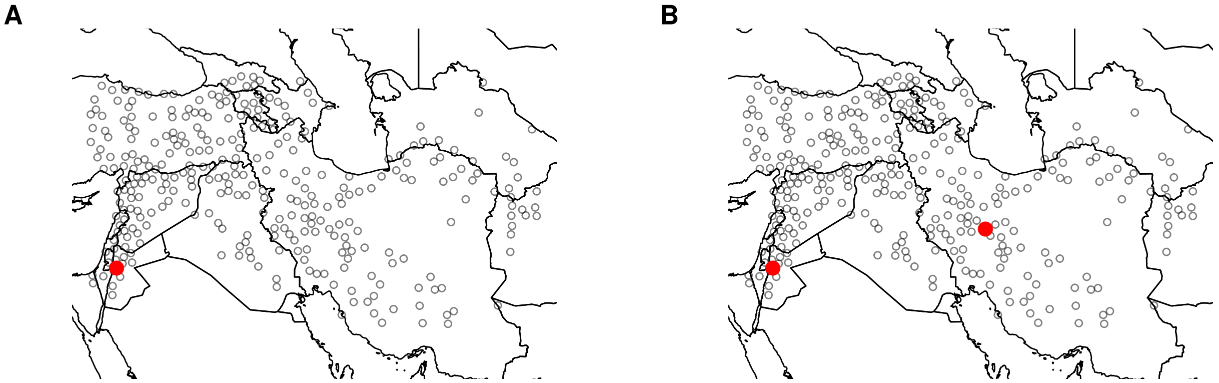

Figure S1 Geographical distribution of sub-populations in two demographic scenarios of SLiM simulation. A. Population expansion from one refugium (1R). B. Population expansion from two refugia (2R). Red dots indicate the starting points of population expansion.

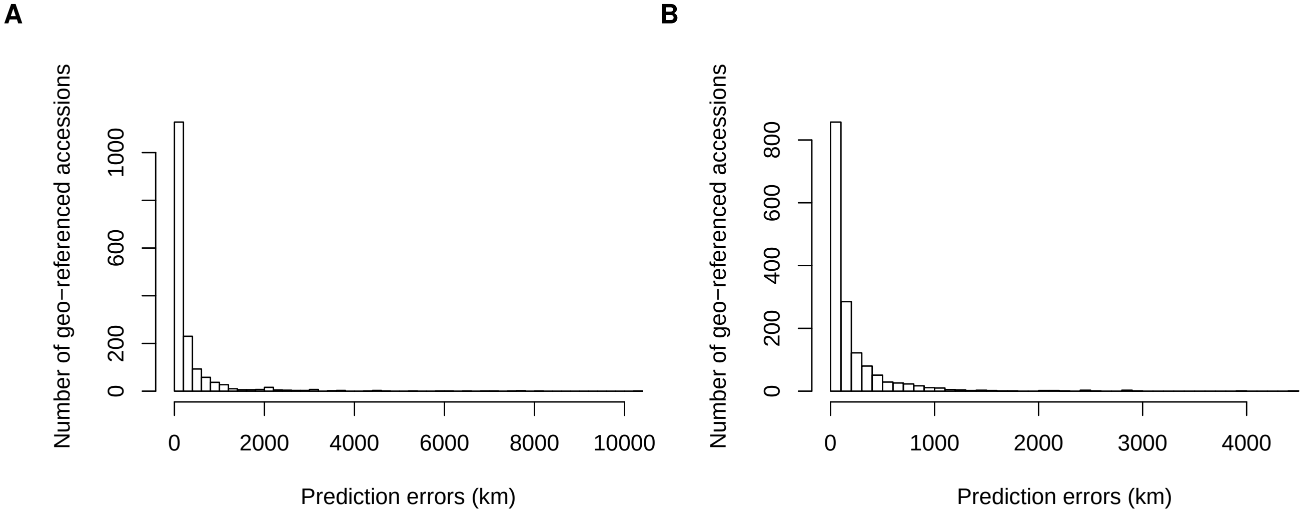

Figure S2 Mean prediction errors in kilo-meters estimated from cross-validation of IPK barley landrace collection. A. Prediction errors of original samples. B. Prediction errors of samples excluding outliers with unusual geo-genetic patterns.

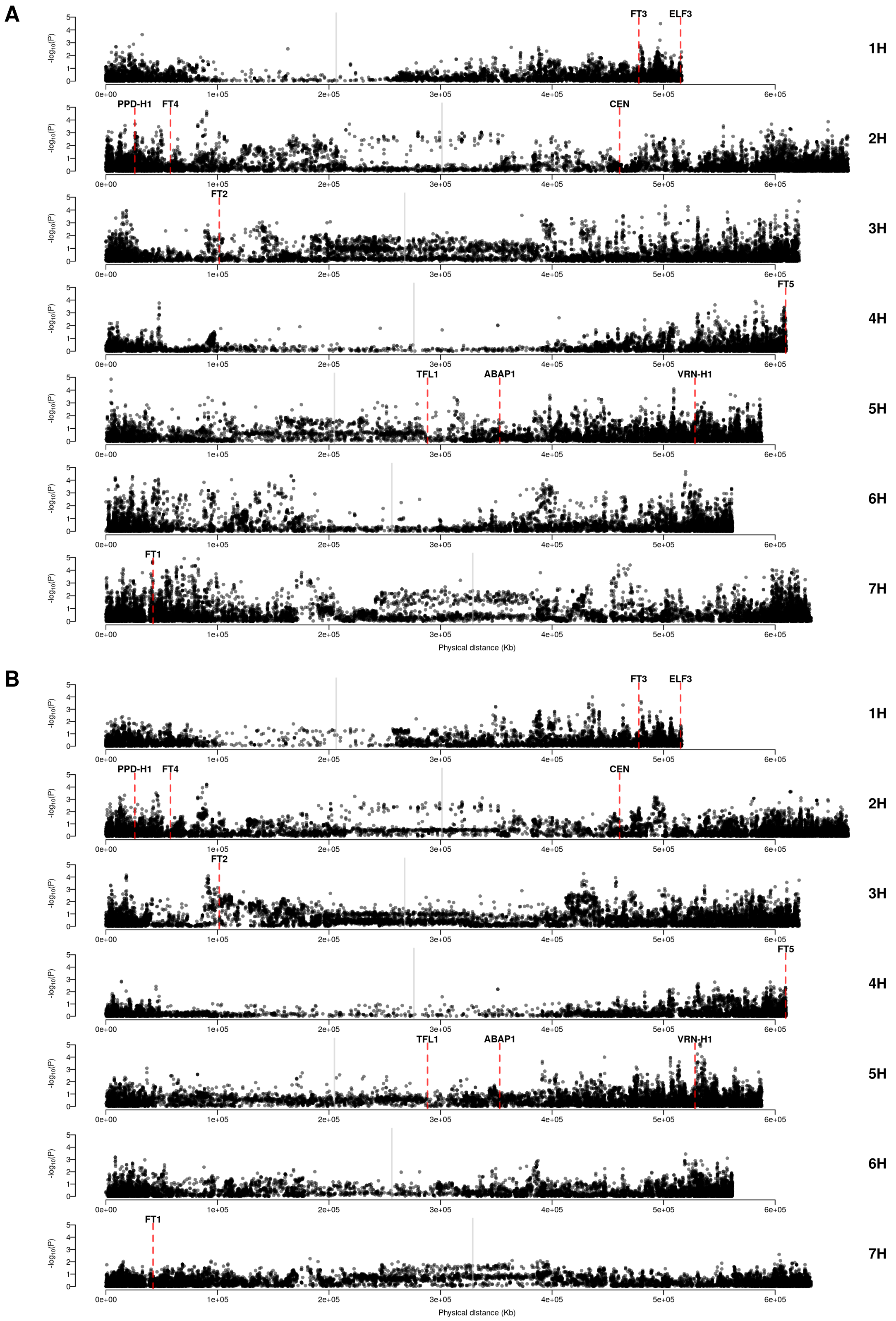

Figure S3 Regular GEA of IPK landraces with the environmental principal component (PC). A. Regular GEA with environmental PC2. B. Regular GEA with environmental PC3. Blue and red horizontal lines are the significant levels of FDR = 0.05 and FDR = 0.01. Grey vertical lines indicate the positions of centromeres. Red dashed lines indicate the positions of flowering time genes.

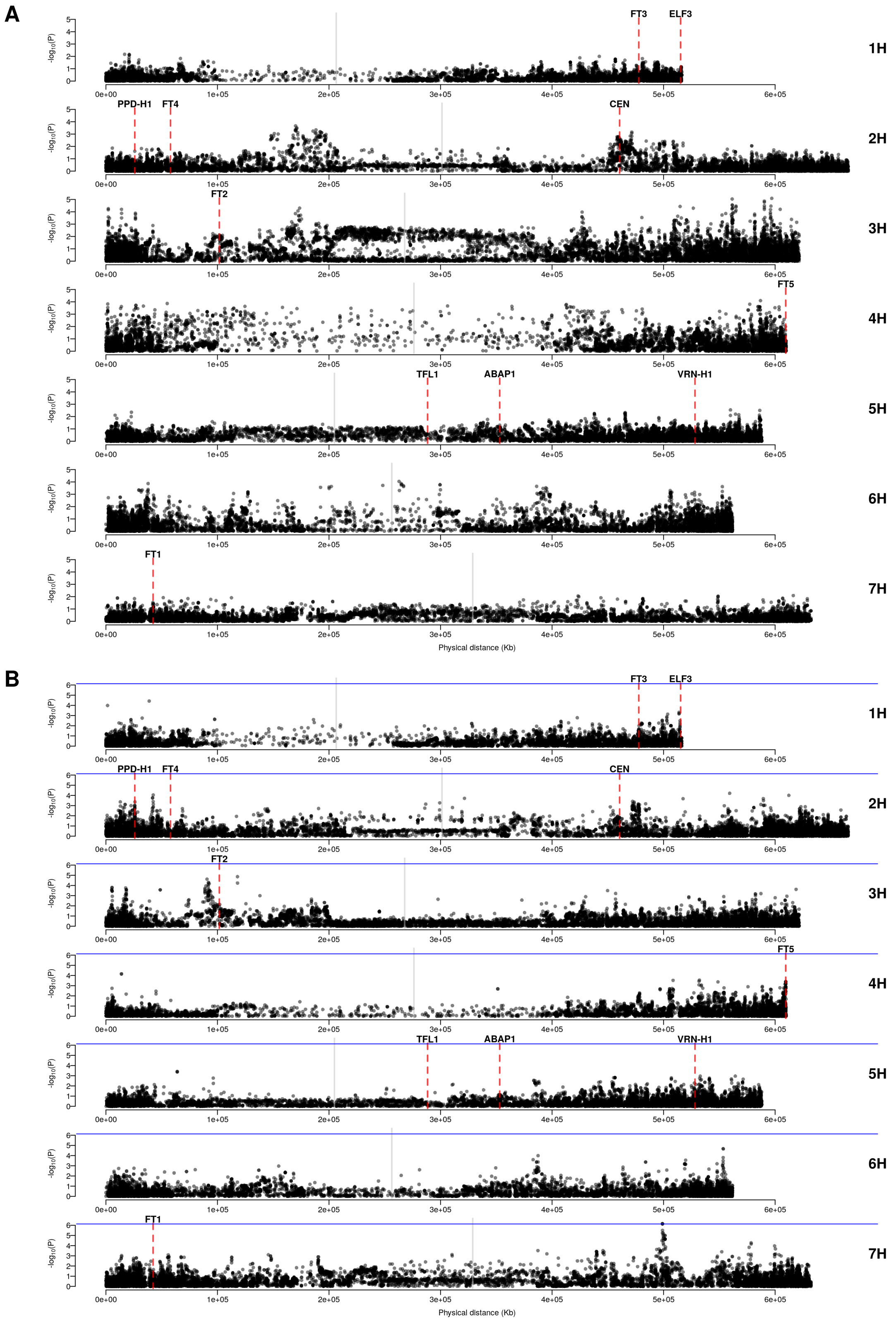

Figure S4 *GEAplus* of IPK landraces with the environmental principal component (PC). A. *GEAplus* with environmental PC1. B. *GEAplus* with environmental PC3. Blue and red horizontal lines are the significant levels of FDR = 0.05 and FDR = 0.01. Grey vertical lines indicate the positions of centromeres. Red dashed lines indicate the positions of flowering time genes.

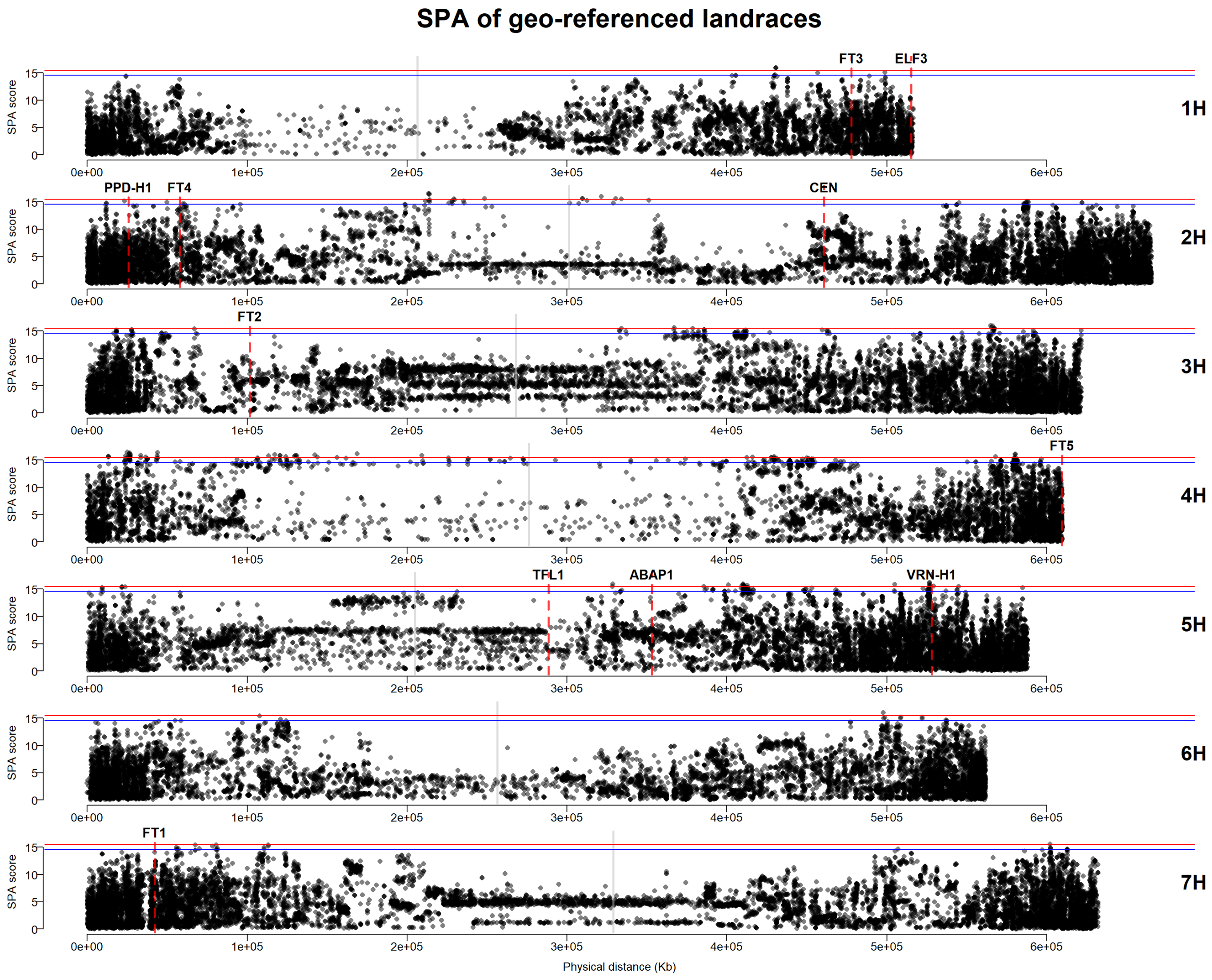

Figure S5 Manhattan plot of SPA conducted using geo-referenced accessions ($N = 1,626$). Blue and red horizontal lines are the empirical cutoff of 0.5% and 0.1%. Grey vertical lines indicate the positions of centromeres. Red dashed lines indicate the positions of flowering time genes.

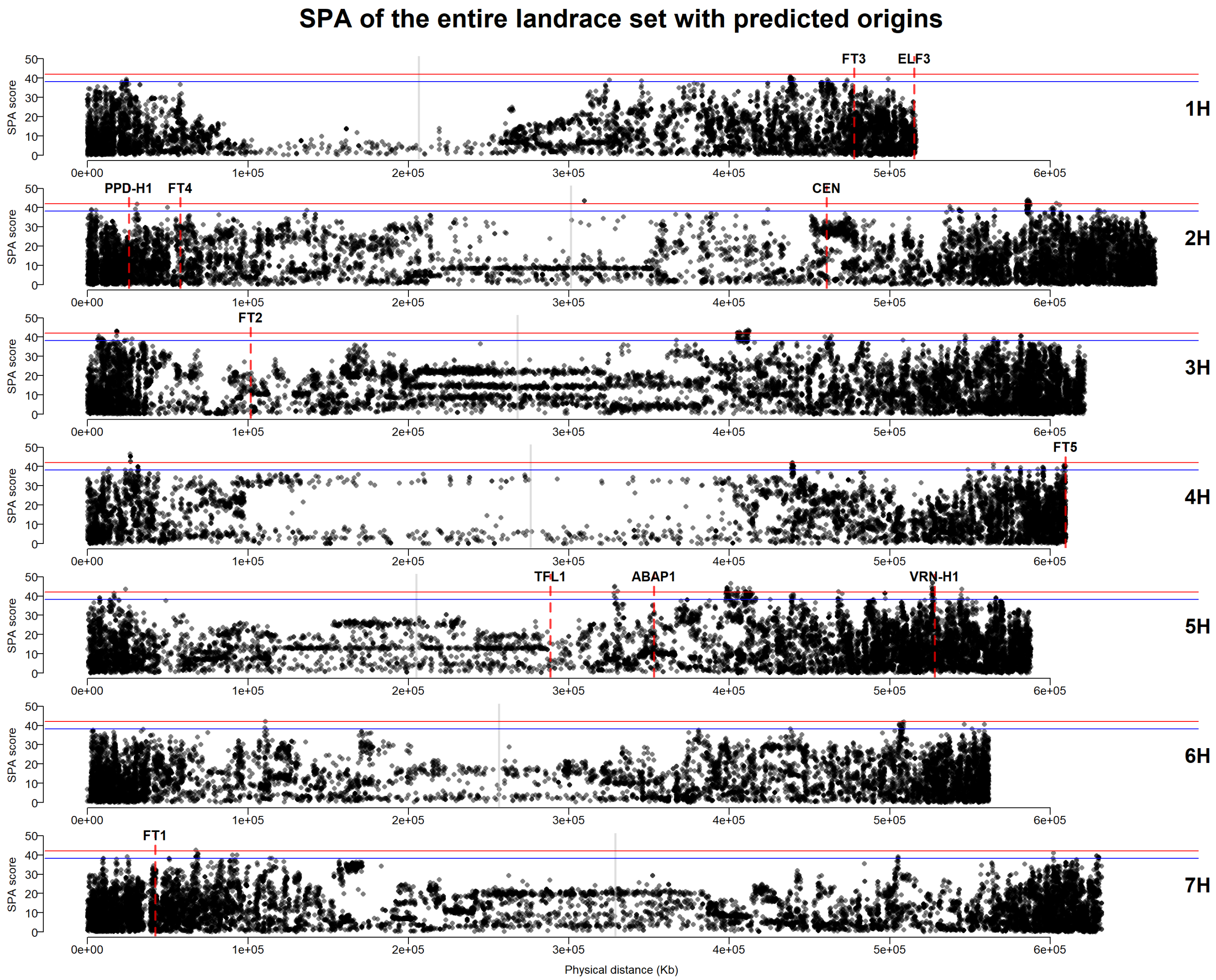

Figure S6 Manhattan plot of SPA conducted using the entire landrace collection with predicted geographical origins ($N = 12,129$). Blue and red horizontal lines are the empirical cutoff of 0.5% and 0.1%. Grey vertical lines indicate the positions of centromeres. Red dashed lines indicate the positions of flowering time genes.

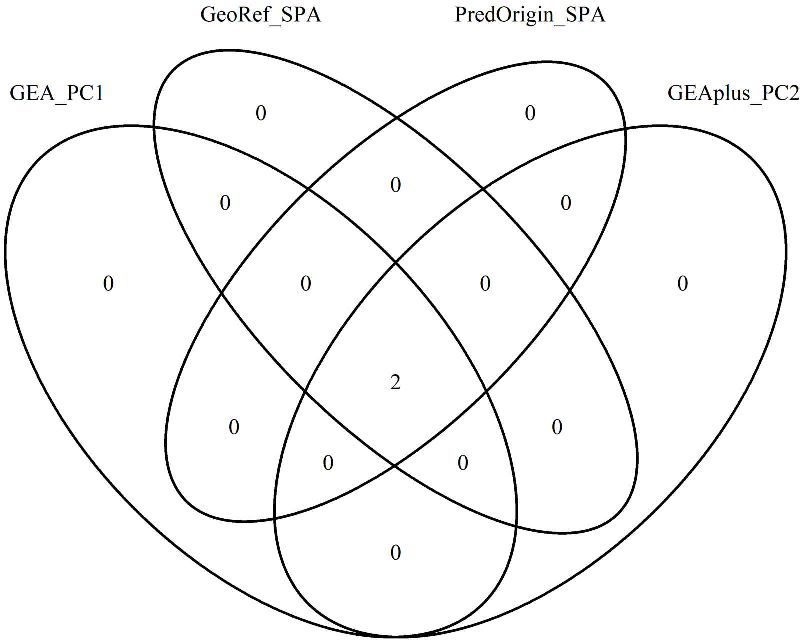

Figure S7 Venn diagram of overlapping significant SNPs. Only *GEA_PC1*, *GEAplus_PC2* and two SPA analyses are included, as these particular approaches had the most significant results.

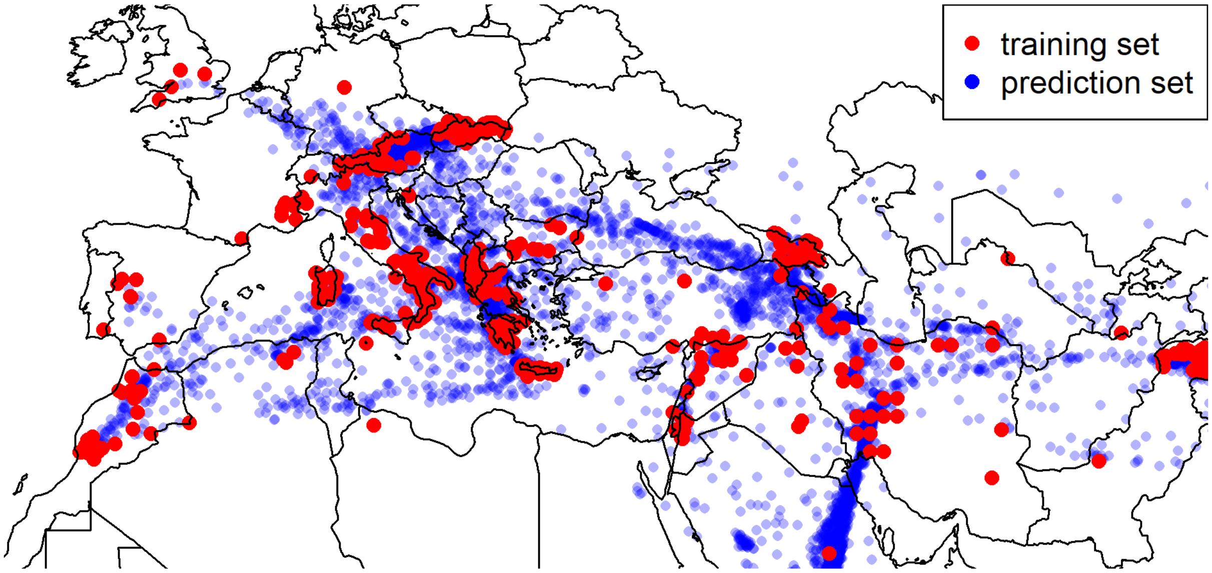

Figure S8 An example map of implausible predictions. It illustrates that landrace accessions of the prediction set (blue dots) are placed in water bodies, such as the Mediterranean Sea, and also in desert regions.
